# Pathological Society of Philadelphia

**Published:** 1877-08

**Authors:** 


### PATHOLOGICAL SOCIETY OF PHILADELPHIA.

THURSDAY EVENING, April 12, 1877.

The President, Dr. H. Lennox Hodge, in the chair.

Intracapsular fracture of femur—Thrombosis of left femoral vein extending to vena cava. By Dr. J. C. Wilson.

The specimen was removed from the body of a gentleman aged sixty-seven, who weighed one hundred and sixty-seven pounds. He fell upon the sidewalk, January 24, and received the full force of the blow upon his left hip. Shortly afterward he was etherized and examined by Dr. Wilson and Dr. J. H. Brinton.

The foot was markedly everted; there was a shortening of the limb of apparently one-half the arc described by the great trochanter in rotation, seemed to be of shorter radius than that of the opposite side; after free movement, crepitus was felt; it was, however, obscure.

A diagnosis of fracture (intracapsular ?) of the neck of the femur was made.

The treatment selected was weight-and-pulley extension, and counter-extension by elevating the foot of the bed. Sand-bags were employed.

Four days afterwards; signs of pelvic cellulitis supervened. These shortly subsided. February 12, the leg became swollen, tense; there was occlusion of the femoral veins, and inflammatory infiltration of the tissues in the line of the great vessels. Fever set in, the temperature rose to 102° Fahr., and remained between $98\frac{1}{4}$° and 102° until he died, March 26. A bedsore had formed over the sacrum. The urine was not albuminous. Liver dulness was enlarged. There were no pulmonary symptoms. Heart-sounds feeble; the first valvular. The pulse feeble; no murmur.

The *post-mortem* examination, made twenty-six hours after death, confirmed the diagnosis of intracapsular fracture. In addition, the heart was found fatty, the lungs slightly adherent but otherwise normal; the liver fatty, and the kidneys cirrhotic. There were evidences of inflammation on the inside of the pelvis opposite the acetabulum, thrombosis of the left femoral and iliac veins extending to the vena cava, as shown in the specimen, with inflammatory infiltration of the tissues surrounding these vessels.

Dr. C. B. Nancrede said that this case of thrombosis of the iliac vein was interesting in connection with the paper he read before the Society a year ago, furnishing an illustration of what he then tried to prove. Here is an injury, a blow over the region of the trochanter major. We know that even a slight blow will produce clotting in the smaller veins, and inflammatory action will be set up by these—the venous clots—in the surrounding tissues. In the venous clot we have present one of the factors necessary for the formation of a clot, viz: the fibrino-plastin, in the shape of the blood-cells. Now let blood containing an excess of the second necessary factor—namely, *fibrinogen*—resulting from the retrograde metamorphosis of tissues, etc., reach the performed clot containing the fibrino-plastin, and it will go on extending until no more blood running from the inflamed spot can reach it. In the case before the Society that point should be about the brim of the pelvis, since normally all the blood from the injured territory would be emptied into the femoral below this point.

Upon examining the specimen, however, we find that it extends still higher? In one of two ways: either blood containing retrograde matter was conveyed from the acetabulum through the pelvic bones, and thus was poured into the external iliac through the internal iliac vein, or, what is still more probable, the clot, irritating the tissues at the brim of the pelvis, owing to the greater pressure due to the sub-jacent bone, established a new depot for the absorption of inflammatory products, and thus extended the clotting until all the venous blood-supply was prevented from reaching the thrombosed vein from the inflamed extremity. This second inflammatory depot is not assumed for the purpose of explanation, but is specially noted by Dr. Wilson in his remarks.

Dr. Hodge said the specimen was interesting from several points of view. First, the fact of embolism being present was of great interest as regards the origin of the clot. A slight cause will produce a clot when least expected. He recalled a case which occurred at the Presbyterian Hospital, in which a leg had been amputated by Dr. T. B. Reed, on account of disease of the ankle-joint. When the patient had almost recovered, and the wound was nearly healed, the opposite thigh became very much swollen and tender over the femoral vein, and there was every reason to suppose a clot had formed in it. This condition presented itself immediately after the man was first lifted from his bed. He recovered.

Another point of interest was in reference to the formation of bony nodules in the capsule around the joint. They do not occur uniformly, and their explanation is more or less difficult. He thought the most reasonable explanation attributed them to fragments of periosteum displaced at the time of fracture, just as the periosteum produces bone after transplantation.

Another point of interest is the apparent injury to the inner surface of the pelvis opposite the acetabulum. He thought this could be accounted for in two different ways. It was possibly due to the direct effects of the original injury, as the acetabulum may be broken by the head of the femur driven forcibly against it. Or this condition may arise from inflammatory action causing softening of the bone.

Still another point, Dr. H. thought, was that, notwithstanding the absence of callus, which is characteristic of injuries in this locality, a great degree of firmness exists. Whether this is due to an attempt at union, or whether this is one of those rare cases of partial fracture of the neck of the femur, can only be determined by sawing the bone.

A final point of interest, he thought, was the preservation of the round ligament, which, from its direction, as the members of the Society may notice, bears out the inference that one of its uses is to aid in the support of the body by bearing a portion of its weight in the erect position.

Dr. Wilson said that the crepitus, the limitation of the arc of a circle described by the trochanter major, together with the shortening, led them to believe the fracture was a complete one.

Dr. Nancrede thought that there was no evidence of any union. The limb had never been stepped upon, and therefore there was none of the ordinary displacement usually seen, and the apparent union was only the result of the interlocking of the fragments.

He thought that if he had taken a few more steps there would have been no donbt about the presence of a complete fracture. Repair was impossible with such interruption to the circulation as existed. The edges of the fragments were as sharp as if broken but yesterday, and, as is well known, the first step towards union should be a proliferation of the connective-tissue cells or the adventitia of the Haversian vessels. This would induce absorption of the earthy matter of the bone, and rounding of the edges of the fragments would ensue.

*A Needle Found post-mortem within the Cranium.*—By Dr. H. Lenox Hodge.—Upon removing the calvaria of a subject in the anatomical rooms of the University of Pennsylvania, a sewing needle of medium size was found lying on the right hemisphere of the brain, nearly parallel to the superior longitudinal sinus, about an inch distant from it, and about an inch and a half behind the fronto-parietal suture. The point and the eye of the needle were both unbroken. The point was directed backwards. The needle was much oxidized, and attached to the arachnoid surface of the dura mater, by old bands of lymph near the larger extremity of the needle.

No history of the cadaver, an adult male, could be obtained.

The needle appears to have given rise to no important changes, and had no apparent connection with the cause of death. The man seems to have died of phthisis.

It is a matter of interest how the needle reached this position. Other methods might be suggested, but it is most probable that it entered the anterior fontanelle during infancy, and thus passed to the place where it was found.

Dr. Hamilton Osgood asked what was the appearance of the needle.

Dr. Hodge replied that it was black and tarnished.

Dr. Sinkler thought it most likely that it had entered the fontanelle during infancy, as by no muscular contraction could it obtain the position in which it was found.

Dr. F. P. Henry said his experience went to show that instead of corrosion of needles long buried in animal tissue, there was an actual addition of new material. A short time ago he had removed with great difficulty a needle from the biceps muscle of a girl. It was three times the thickness of an ordinary needle, very rough and uneven, and covered with a hard, mineral-like depost, to which was owing the increase in thickness.

Dr. Wilson recited the case of a sewing-girl who was said to have swallowed a paper of needles, many of which were removed from different parts of the body. These were all smooth, but blackened and tarnished. He had recently removed from the foot of a boy a needle which had been imbedded for four months. It was simply tarnished.

Dr. Sinkler had removed a needle from a foot after it had been there imbedded for three months. It was smooth and blackened, but not corroded. He placed it in his pocket-book, and on examining it after a few weeks he observed that the rusting process had taken place.

Dr. Richard A. Clemann said that he had made use of the fact that a needle, after being imbedded in tissue for a certain length of time, becomes tarnished. He had extracted a fragment of needle and was anxious to determine whether it was all that had entered the foot. The broken end was tarnished. He fractured the needle, and observing that the fractured ends presented the usual steel-like luster, he concluded that he had removed the whole fragment. Had he broken it off the fractured end of the removed portion would have been bright.

*Gangrene of the Epiglottis.*—By Dr. Carl Seiler.—The patient, a man presented himself at the infirmary of the German Hospital. Nearly one-half of the larynx had sloughed off in two weeks. The odor was so horrible that a laryngoscopic examination was almost impossible. The man was recommended to go to a general hospital, and has not been heard of since.—*Medical Times.*

